# The associations of leukocyte telomere length and intermediary cardiovascular phenotype with adverse cardiovascular outcomes in the white population

**DOI:** 10.1038/s41598-024-64997-3

**Published:** 2024-06-17

**Authors:** Ho-Gi Chung, Pil-Sung Yang, Seunghoon Cho, Eunsun Jang, Daehoon Kim, Hee Tae Yu, Tae-Hoon Kim, Jae-Sun Uhm, Jung-Hoon Sung, Hui-Nam Pak, Moon-Hyoung Lee, Boyoung Joung

**Affiliations:** 1 Division of Cardiology, Department of Internal Medicine, Severance Cardiovascular Hospital, Yonsei University College of Medicine, 50-1 Yonsei-ro, Seodaemun-gu, Seoul, 03722 South Korea; 2 Department of Cardiology, CHA Bundang Medical Center, CHA University, Seongnam, South Korea

**Keywords:** Leukocyte telomere length, Sudden cardiac death, Coronary event, Heart failure, Cardiac magnetic resonance imaging, Electrocardiogram, Cardiology, Medical research

## Abstract

The evidence about the associations of leukocyte telomere length (LTL) and intermediary cardiovascular phenotypes with adverse cardiovascular outcomes is inconclusive. This study assessed these relationships with cardiovascular imaging, electrocardiography, and the risks of sudden cardiac death (SCD), coronary events, and heart failure (HF) admission. We conducted a cross-sectional analysis of UK Biobank participants enrolled between 2006 and 2010. LTL was measured using quantitative polymerase chain reactions. Electronic health records were used to determine the incidence of SCD, coronary events, and HF admission. Cardiovascular measurements were made using cardiovascular magnetic resonance imaging and machine learning. The associations of LTL with SCD, coronary events, and HF admission and cardiac magnetic resonance imaging, electrocardiogram parameters of 33,043 and 19,554 participants were evaluated by multivariate regression. The median (interquartile range) follow-up period was 11.9 (11.2–12.6) years. Data was analyzed from January to May 2023. Among the 403,382 white participants without coronary artery disease or HF, 181,637 (45.0%) were male with a mean age of 57.1 years old. LTL was independently negatively associated with a risk of SCD (LTL third quartile vs first quartile: hazard ratio [HR]: 0.81, 95% confidence interval [CI]: 0.72–0.92), coronary events (LTL third quartile vs first quartile: HR: 0.88, 95% CI: 0.84–0.92), and HF admission (LTL fourth quartile vs first quartile: HR: 0.84, 95% CI: 0.74–0.95). LTL was also independently positively associated with cardiac remodeling, specifically left ventricular mass index, left-ventricular-end systolic and diastolic volumes, mean left ventricular myocardial wall thickness, left ventricular stroke volume, and with electrocardiogram changes along the negative degree of T-axis. Cross-sectional study results showed that LTL was positively associated with heart size and cardiac function in middle age, but electrocardiography results did not show these associations, which could explain the negative association between LTL and risk of SCD, coronary events, and HF admission in UK Biobank participants.

## Introduction

The incidence of many cardiovascular diseases increases with chronological age, including sudden cardiac death (SCD), coronary heart disease, and heart failure (HF)^1,2^. Telomeres, which contribute to cell genomic stability, are non-coding repetitive TTAGGG nucleoprotein structures located at the end of each chromosome^3^. Telomere length is used to define biological age because it shortens after every cell division^4,5^. When telomere length shortens to a critical value, which acts as a protective cap for the chromosome, cell senescence occurs^6^. Telomere length might be related to cardiovascular disease incidence.

Telomere length can be measured in terms of leukocyte telomere length (LTL), which is associated with other tissues’ telomere length^7^. LTL is negatively associated with the risk of cardiovascular disease, such as coronary artery disease, myocardial infarction, and HF^8,9^. It is not clear why this association exists between LTL and cardiovascular diseases that can cause SCD, such as coronary events and HF admission. LTL shortening may reflect changes in coronary artery atherosclerosis, myocardium structure and function, and electrocardiogram (ECG) parameters^10,11^. The evidence about the association of LTL and intermediary cardiovascular phenotypes with adverse cardiovascular outcomes is inconclusive. Thus, this study assessed the associations of LTL with cardiovascular imaging and ECG phenotypes and the risk of SCD, coronary events, and HF admission.

We hypothesized that LTL is associated with intermediary cardiovascular phenotypes as defined by cardiac magnetic resonance (CMR) and ECG parameters, which would explain the incidences of SCD, coronary events, and HF admission. Using UK Biobank registries, we determined how LTL was associated with CMR and ECG parameters and clinical events, namely SCD, coronary events, and HF admission.

## Methods

### UK Biobank cohort participants

The UK Biobank contains data about 502,421 participants aged 40–69 years old collected from 2006–2010 at 22 assessment centers in England, Scotland, and Wales^12,13^. The UK Biobank contains health-related information about participants that was collected by comprehensive questionnaires, face-to-face interviews, physical measurements, imaging, and blood sampling. Participants were dropped out of the UK Biobank registry if they were not willing to be involved during follow-up.

**Figure 1:**
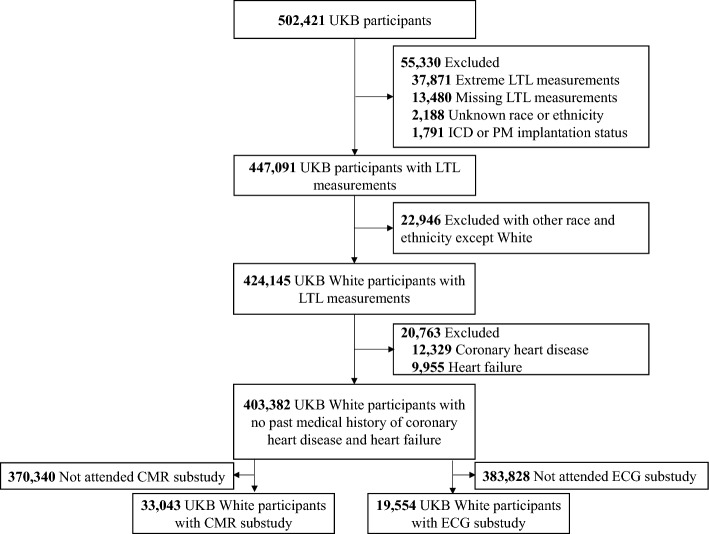
Sample selection flowchart. *CMR* cardiovascular magnetic resonance imaging, *ECG* electrocardiogram, *ICD* implantable cardioverter defibrillator, *LTL* leukocyte telomere length, *PM* pacemaker, *UKB* UK Biobank.

Of the participants, we excluded 37,871 with extreme LTLs and LTLs shorter than the 1–1.5 interquartile range and longer than 3 + 1.5 interquartile range; 13,480 participants with missing LTLs or white blood cell counts; 1791 participants with implantable cardioverter defibrillators or implanted pacemakers; and 2188 participants with an unknown race or ethnicity^14^. Since there were some reports that leukocyte telomere length is related to ethnicity, and considering most of the participants in the UK Biobank registry are white, we restricted to white race. Among the remaining 447,091 participants, 403,382 white participants with no past medical history of coronary heart disease or HF were included in the study sample (Fig. 1). Of these participants, 33,043 were used for the CMR substudy and 19,554 were used for the ECG substudy. This study received ethical approval from North West—Haydock Research Ethics Committee on June 18, 2021 (REC reference: 21/NM/0157) and was conducted under application No. 77793. Informed consent was obtained from all participants. All of our research methods followed the relevant guidelines and regulations. This study followed the Strengthening the Reporting of Observational Studies in Epidemiology (STROBE) reporting guideline for cohort studies (Supplementary Table 1).

### LTL measurement

The LTL measurement, quality check, adjustment, and Z-score standardization methods were conducted as described in previous studies^15^. In brief, LTL was measured using the multiplex quantitative polymerase chain reaction (PCR) method. LTL was measured by dividing the number of leukocyte telomere repeat copies (T) by the number of single-copy genes (S). In each reaction, the amount of T and S were measured and calculated relative to a calibrator sample of pooled DNA from 20 participants. LTL was adjusted for enzyme, PCR machine, primer, operator, temperature, humidity, primer*PCR machine, primer*operator, and A260/A280. LTLs were measured and reported by researchers at the University of Leicester, England. Standardized Z-scores of the log*e*-adjusted T/S ratio were used (UK biobank data field code: 22192).

### Definition of covariates and primary and secondary outcomes

Definitions used for defining the baseline comorbidities are listed in Supplementary Table 2. The primary outcome was defined as SCD incidence. The secondary outcome was defined as the incidences of coronary events and HF admission. SCD was defined as the presence of ICD-10 code I46 or I49.0 with a code date after the date on which the baseline assessment was conducted in the Hospital Episode Statistics-Admitted Patient Care (HES-APC, England), the Patient Episode Database for Wales-Admitted Patient Care (PEDW-APC, Wales), the Scottish Morbidity Records 01 (SMR01), and the Scottish Morbidity Records 04 (SMR04). Coronary events were defined as the presence of an ICD code related to death by coronary artery disease or myocardial infarction; any acute myocardial infarction, which was defined as ICD-10 code I21 or I22 in HES-APC, PEDW-APC, SMR01, or SMR04; or any coronary revascularization, defined as K40–51 or K75 in HES-APC, PEDW-APC, SMR01, or SMR04. HF admission was defined as the presence of ICD-10 code I11.0 or I50 or I97.1 as a primary diagnosis in HES-APC, PEDW-APC, SMR01, or SMR04. All participants’ data in the UK Biobank registry received updates related to follow-up health information and diagnosis included in the National Health Service. Expert adjudicated and ascertained follow-up health information and diagnosis which provides the accuracy and reliability of the UK Biobank ICD codes related to SCD, coronary events, and HF admission^16,17^.

### Derivation of ECG parameters

ECG measurement and analysis were done as described in previous studies^18^. In UK biobank registry, GE CardioSoft software was used to detect arrhythmias and measure the QRS duration, QT interval, QTc interval, RR interval, QRS-axis, and T-axis in ECG data. The mean ± standard deviation (SD) was measured for each of these parameters 7.9 ± 1.5 years after the participants’ baseline visit.

### Derivation of CMR parameters

CMR measurement and analysis were conducted as described in previous studies^18–22^. Imaging was conducted for participants whose data was collected at the Reading, Stockport, Newcastle, and Bristol sites. First, readers who received training and followed a standardized quality control procedure manually analyzed 5000 CMR scans by segmenting all four cardiac chambers at labs in London and Oxford. However, manual analysis is time-consuming and prone to subjective errors, so Bai et al. demonstrated that an automated CMR image analysis method had a similar level of performance as expert analysis using 4875 manually analyzed CMR images^21^. Thus, a machine learning algorithm was used to segment the left atrium, left ventricle (LV), right atrium, right ventricle, and LV’s myocardium. The LV stroke volume was calculated by subtracting the LV-end systolic volume from the LV-end diastolic volume. The left ventricular ejection fraction was calculated by dividing the LV stroke volume by the LV-end diastolic volume. LV mass divided by body surface area at the time of MRI was scanned was defined as LV mass index. LV-end systolic volume, LV-end diastolic volume, mean LV myocardial wall thickness, LV circumferential global strain, LV radial global strain, and LV longitudinal global strain were derived by the machine learning algorithm. The mean ± SD of these parameters was obtained 8.8 ± 1.7 years after the baseline visit.

### Statistical analysis

Participants’ baseline characteristics were compared by quartiles of the Z-scores of the log_*e*_-transformed adjusted LTL T/S ratio and summarized as mean ± SD for continuous variables and counts and percentages for categorical variables. The time to events was defined as the number of days that passed from the date on which participants enrolled in the UK Biobank registry to the date when a medical event occurred, which was defined as the study endpoint. The primary outcome was SCD incidence. The secondary outcomes were the incidences of coronary events and HF admission. The hazard ratio (HR) and 95% confidence interval (CI) were obtained using multivariable Cox regression analysis adjusting for age, sex, height, weight, hypertension, diabetes, dyslipidemia, and ever smoked history using the lowest quartile of the Z-score of the log*e*-transformed adjusted LTL T/S ratio as the reference category for both primary and secondary outcomes. Schoenfeld residuals were used to assess proportional hazard assumptions for primary and secondary outcomes. Risk of SCD was additionally adjusted by incident coronary events and HF admission as time-dependent variable. Fine-Grey sub-distribution hazard model was used to perform competing risk multivariable regression, wherein all-cause-mortality was set as the competing risk for SCD. Subgroup analysis was done for the primary outcome stratified by age (age < 65 versus age ≥ 65), sex, hypertension, diabetes, dyslipidemia, atrial fibrillation, chronic kidney disease or end-stage renal disease by using interaction terms. The β coefficient and 95% CI for CMR and ECG parameters were adjusted by age, sex, height, weight, systolic blood pressure, diabetes, dyslipidemia, ever smoked history, and total metabolic equivalent of task minutes per week. We used restricted cubic spline curves to examine the association of LTL, a continuous variable, with incidences of SCD, coronary events and HF admission. Fitted restricted cubic spline curves for the HRs of SCD, coronary events and HF admission associated with the Z-score of the log*e*-transformed adjusted T/S ratios were adjusted by age, sex, height, weight, hypertension, diabetes, dyslipidemia, and ever smoked history. 0.6% missing data in the primary and secondary outcome analysis, 2.9% missing data in the CMR parameter analysis, and 0.2% missing data in the ECG parameter analysis related to lifestyle factor and anthropometrics were dropped. Statistical significance was defined as a two-tailed p-value lower than 0.05. Statistical analyses were conducted by using R software version 4.3.1 (R Foundation for Statistical Computing, Vienna, Austria).

### Ethics declarations

This study received ethical approval from North West—Haydock Research Ethics Committee on June 18, 2021 (REC reference: 21/NM/0157) and was conducted under application No. 77793. Informed consent was obtained from all participants. All of our research methods followed the relevant guidelines and regulations.

## Results

### Study population

The baseline characteristics of the 403,382 UK Biobank registry participants, 33,043 CMR study participants, and 19,554 ECG study participants are presented in Table 1. Baseline characteristics between non-CMR and CMR group are presented in Supplementary Table 3 and baseline characteristics between non-ECG and ECG group are presented in Supplementary Table 4. Participants were grouped into standardized Z-scores of the log*e*-transformed adjusted LTL T/S ratio quartiles: shortest (less than − 0.656), second (− 0.656 to − 0.022), third (− 0.022 to 0.656), and longest (greater than 0.656), which contained 100,846 (25.0%), 100,844 (25.0%), 100,846 (25.0%), and 100,846 (25.0%) participants, respectively. Among the 403,382 participants (male: 45.0%, mean age ± SD: 57.1 ± 8.0), participants in the higher LTL quartiles were more likely to be younger and female and less likely to have ever smoked history and cardiovascular or cerebrovascular comorbidities.

**Table 1:** Study cohort characteristics.

Cohort characteristics	Full cohort	LTL quartile				P value
		1st	2nd	3rd	4th	
Age at telomere visit, y	57.1 ± 8.0	59.1 ± 7.6	57.7 ± 7.9	56.6 ± 8.0	55.2 ± 8.1	< 0.001
Sex, no. (%)						
Female	221,745 (55.0)	49,939 (49.5)	53,580 (53.1)	57,053 (56.6)	61,173 (60.7)	< 0.001
Male	181,637 (45.0)	50,907 (50.5)	47,264 (46.9)	43,793 (43.4)	39,673 (39.3)	
Height, cm	168.7 ± 9.3	168.9 ± 9.3	168.7 ± 9.3	168.6 ± 9.3	168.4 ± 9.2	< 0.001
Weight, kg	77.9 ± 15.8	78.8 ± 15.7	78.3 ± 15.8	77.8 ± 15.8	76.8 ± 15.7	< 0.001
Systolic BP, mmHg	138.2 ± 18.7	139.8 ± 18.9	138.8 ± 18.7	137.7 ± 18.6	136.6 ± 18.6	< 0.001
Diastolic BP, mmHg	82.4 ± 10.1	82.6 ± 10.1	82.5 ± 10.1	82.3 ± 10.2	82.0 ± 10.2	< 0.001
Heart rate, beats/min	69.7 ± 11.6	70.0 ± 11.8	69.7 ± 11.7	69.6 ± 11.5	69.4 ± 11.4	< 0.001
Hypertension, %	26.8	29.8	27.7	26.1	23.8	< 0.001
Diabetes, %	4.3	5.4	4.5	4.0	3.4	< 0.001
Dyslipidemia, %	12.7	15.0	13.6	12.0	10.4	< 0.001
Ever smoked, %	60.2	62.5	60.9	59.7	58.8	< 0.001
Physical activity (total MET min per wk)	2678.8 ± 2722.2	2730.2 ± 2769.3	2701.8 ± 2754.6	2660.7 ± 2715.3	2623.5 ± 2648.2	< 0.001
WBC, count/*μL*	6900 ± 2000	7000 ± 2100	6900 ± 1900	6800 ± 2000	6800 ± 2000	< 0.001
Ischemic stroke or TIA, %	0.7	1.0	0.8	0.7	0.5	< 0.001
PAOD, %	0.2	0.2	0.2	0.1	0.1	< 0.001
COPD, %	0.2	0.3	0.2	0.2	0.1	< 0.001
CKD or ESRD, %	0.7	1.0	0.8	0.6	0.6	< 0.001
AF, %	1.0	1.3	1.0	1.0	0.8	< 0.001
VT history, %	0.2	0.3	0.3	0.2	0.2	< 0.001
BP medication, %	18.9	21.5	19.7	18.1	16.2	< 0.001
Statin, %	13.4	16.1	14.2	12.5	10.6	< 0.001
Antiplatelet, %	11.9	14.1	12.6	11.1	9.7	< 0.001
CMR substudy characteristics*						
Age at telomere visit, y	55.5 ± 7.4	57.1 ± 7.3	55.9 ± 7.3	55.1 ± 7.4	53.8 ± 7.4	< 0.001
Year at imaging visit after telomere visit, y	8.8 ± 1.7	8.7 ± 1.7	8.8 ± 1.7	8.9 ± 1.7	8.9 ± 1.7	< 0.001
CMR parameters						
LV-end diastolic volume, mL	147.8 ± 33.5	148.0 ± 33.5	148.0 ± 34.0	147.0 ± 33.4	147.5 ± 33.3	0.760
LV-end systolic volume, mL	60.3 ± 19.0	60.6 ± 19.3	60.3 ± 19.1	60.3 ± 18.8	59.9 ± 18.7	0.161
LV stroke volume, mL/beat	87.5 ± 19.2	87.4 ± 19.1	87.7 ± 19.4	87.4 ± 19.3	87.6 ± 19.1	0.596
LVEF, %	59.6 ± 6.1	59.5 ± 6.2	59.7 ± 6.0	59.5 ± 6.1	59.8 ± 5.9	0.011
LVMI, g/m^2^	45.7 ± 8.5	45.9 ± 8.5	45.9 ± 8.7	45.6 ± 8.3	45.5 ± 8.4	0.001
Mean LV myocardial wall thickness, mm	5.7 ± 0.8	5.7 ± 0.8	5.7 ± 0.8	5.7 ± 0.8	5.6 ± 0.8	< 0.001
LV circumferential global strain, %	− 22.3 ± 3.4	− 22.2 ± 3.4	− 22.3 ± 3.4	− 22.3 ± 3.4	− 22.4 ± 3.3	0.011
LV radial global strain, %	45.1 ± 8.3	45.0 ± 8.4	45.2 ± 8.3	45.1 ± 8.4	45.2 ± 8.1	0.221
LV longitudinal global strain, %	− 18.5 ± 2.8	− 18.4 ± 2.8	− 18.5 ± 2.8	− 18.5 ± 2.8	− 18.6 ± 2.8	0.013
ECG substudy characteristics^†^						
Age at telomere visit, y	55.5 ± 7.5	57.2 ± 7.2	55.9 ± 7.4	55.2 ± 7.5	53.8 ± 7.4	< 0.001
Year at ECG visit after telomere visit, y	7.9 ± 1.5	7.8 ± 1.5	7.8 ± 1.5	7.9 ± 1.5	7.9 ± 1.5	< 0.001
ECG parameters						
QRS duration, ms	88.7 ± 13.5	88.9 ± 14.0	89.2 ± 13.5	88.6 ± 13.5	88.0 ± 12.8	< 0.001
QT interval, ms	418.3 ± 31.5	417.8 ± 31.4	417.5 ± 31.9	418.7 ± 30.8	419.0 ± 31.9	0.049
QTc interval, ms	420.3 ± 25.2	420.1 ± 25.0	420.3 ± 25.8	420.2 ± 24.8	420.5 ± 25.2	0.805
RR interval, ms	996.7 ± 164.8	995.5 ± 164.5	992.3 ± 160.8	999.4 ± 162.9	999.6 ± 170.9	0.085
QRS-axis, degree	27.7 ± 34.8	26.3 ± 35.3	27.4 ± 35.0	27.8 ± 34.3	29.3 ± 34.5	< 0.001
T-axis, degree	39.3 ± 30.1	39.9 ± 31.7	39.4 ± 29.4	38.7 ± 29.6	39.1 ± 29.7	0.318

Values are presented as mean ± standard deviation or number (%).

*AF* atrial fibrillation, *BP* blood pressure, *CKD* chronic kidney disease, *CMR* cardiovascular magnetic resonance imaging, *COPD* chronic obstructive pulmonary disease, *ECG* electrocardiogram, *ESRD* end stage renal disease, *LTL* leukocyte telomere length, *LV* left ventricle, *LVEF* left ventricle ejection fraction, *LVMI* left ventricle mass index, *MET* metabolic equivalent of task, *NA* not applicable, *PAOD* peripheral artery occlusive disease, *SD* standard deviation, *TIA* transient ischemic attack, *VT* ventricular tachycardia, *WBC* white blood cell.

*33,043 participants were involved in CMR substudy.

^†^19,554 participants were involved in ECG substudy.

### Longitudinal association between LTL and SCD

**Figure 2:**
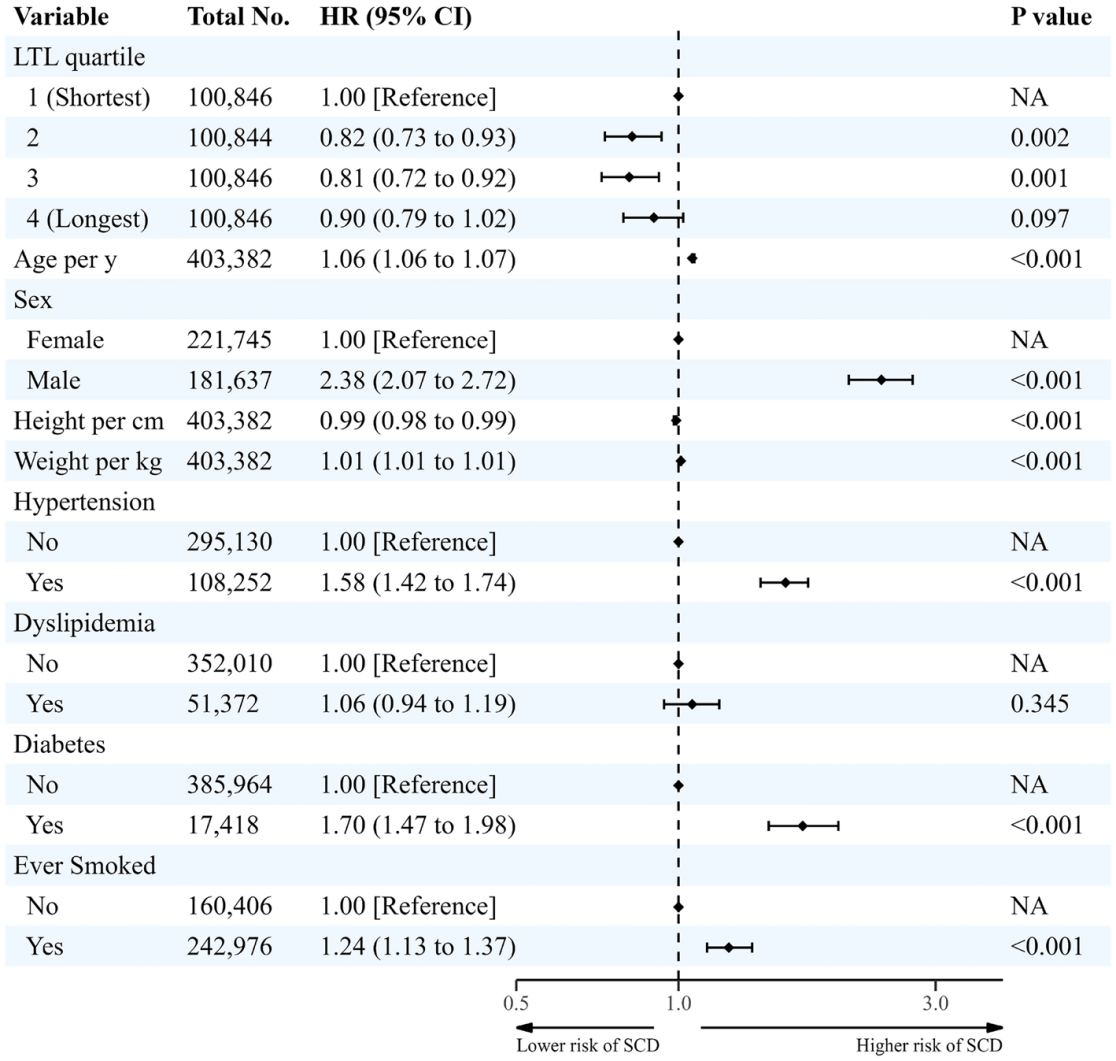
Longitudinal association between leukocyte telomere length and incidence of sudden cardiac death in white participants. *CI* confidence interval, *HR* hazard ratio, *LTL* leukocyte telomere length, *NA* not applicable, *SCD* sudden cardiac death. Cox proportional hazards analysis was adjusted for age, sex, height, weight, hypertension, diabetes, dyslipidemia and ever smoked history.

Multivariable Cox regression analysis results for the association between LTL and SCD incidence are presented in Fig. 2 and Supplementary Table 5. During the median follow-up period of 11.9 years (interquartile range: 11.2–12.6 years), SCD occurred in 1889 participants with an incidence rate of 0.04 per 100 person-years (PYRs). Cox proportional hazards analysis showed that LTL was negatively associated with SCD incidence. Compared with the shortest LTL quartile group (0.05 per 100 PYRs), the incidence rate and adjusted SCD risk were significantly lower in participants in the second (0.04 per 100 PYRs, HR: 0.82, 95% CI: 0.73–0.93; p = 0.002) and third quartiles (0.03 per 100 PYRs, HR: 0.81, 95% CI: 0.72–0.92, p = 0.001). However, the adjusted SCD of the longest quartile was not statistically significantly different from that of the shortest quartile (0.03 per 100 PYRs, HR: 0.90, 95% CI: 0.79–1.02, p = 0.097). Every 1 SD increase in LTL was associated with a lower SCD risk (HR: 0.92, 95% CI: 0.88–0.97, p < 0.001).

Results did not change when additionally adjusted by incident coronary event and HF admission as a time-dependent variable. SCD risk were significantly lower in participants in the second (HR: 0.85, 95% CI: 0.75–0.96, p = 0.009) and third quartiles (HR: 0.85, 95% CI: 0.75–0.97, p = 0.014) than the shortest quartile group. However, the adjusted SCD of the longest quartile was not statistically significantly different from that of the shortest quartile (HR: 0.97, 95% CI: 0.85–1.11, p = 0.666). Incident coronary event and HF admission were statistically significantly related to SCD (HR: 32.29, 95% CI: 29.21–35.69, p < 0.001).

Results did not also change in the Fine-Grey competing risk multivariable regression model. Adjusted SCD risk were significantly lower in participants in the second (HR: 0.83, 95% CI: 0.73–0.94, p = 0.002) and third quartiles (HR: 0.82, 95% CI: 0.73–0.93, p = 0.002) than the shortest quartile group. However, the adjusted SCD of the longest quartile was not statistically significantly different from that of the shortest quartile (HR: 0.90, 95% CI: 0.80–1.03, p = 0.127).

Risk factors related to cardiovascular disease were associated with SCD, namely hypertension (HR: 1.58, 95% CI: 1.42–1.74, p < 0.001), diabetes (HR: 1.70, 95% CI: 1.47–1.98, p < 0.001), and ever smoked history (HR: 1.24, 95% CI: 1.13–1.37. p < 0.001). The epidemiological factors of sex, age, height, and weight were also associated with SCD incidence (Fig. 2).

Figure 5a shows the association between SCD incidence and LTL using the restricted cubic spline curve. The association between LTL and SCD had a U-shape with a nonlinear p of 0.028. The slope was steeply negative below a standardized Z-score of the log*e*-adjusted LTL T/S ratio of 0, then reached a plateau and became positive above 0.

### Longitudinal associations of coronary events and HF admission with LTL

**Figure 3:**
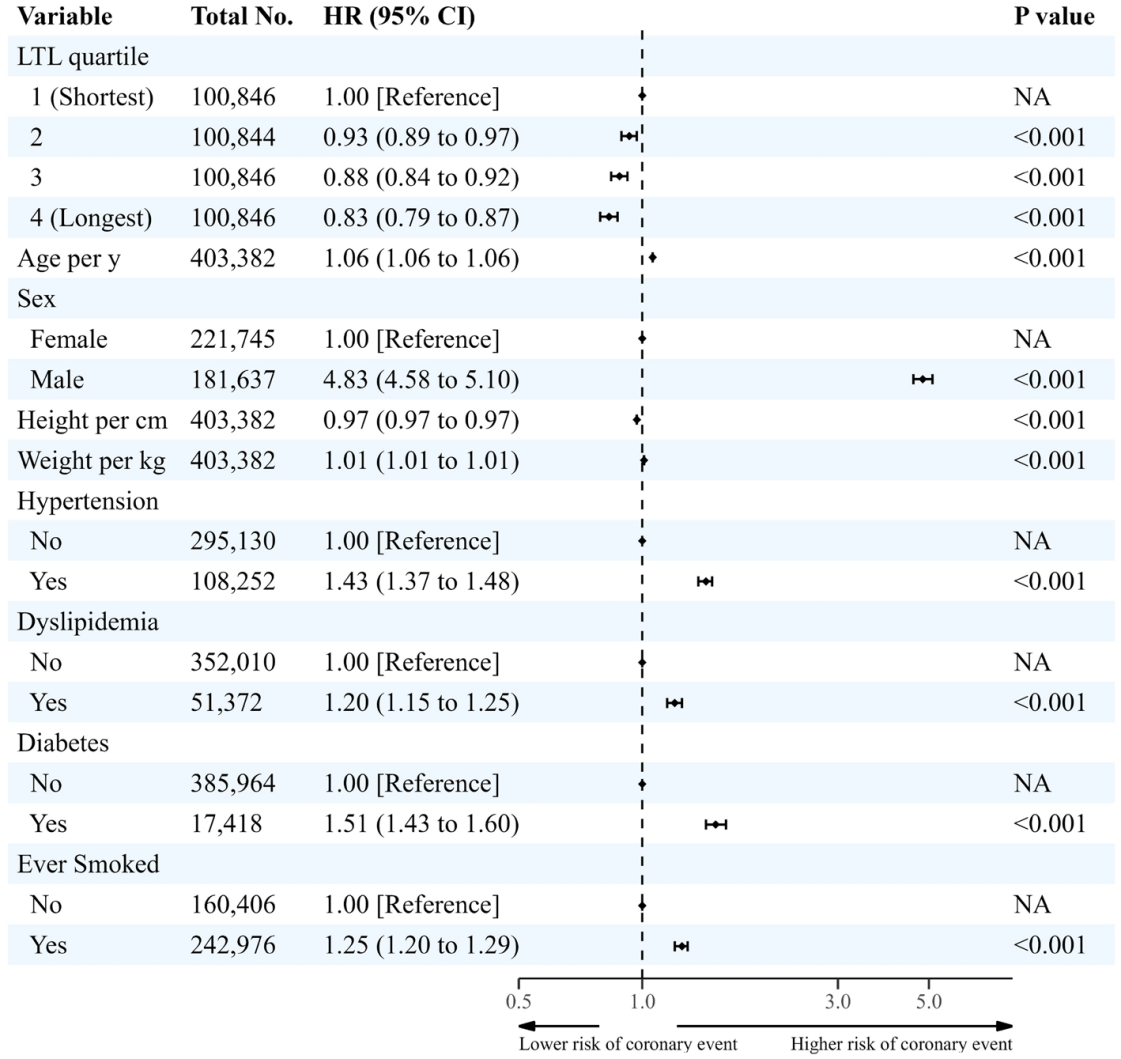
Longitudinal association between leukocyte telomere length and incidence of coronary event in white participants. *CI* confidence interval, *HR* hazard ratio, *LTL* leukocyte telomere length, *NA* not applicable. Coronary event was a composite of death by coronary artery disease or myocardial infarction, any acute myocardial infarction, any coronary revascularization. Cox proportional hazards analysis was adjusted for age, sex, height, weight, hypertension, diabetes, dyslipidemia and ever smoked history.

The mean ± SD time to event when a medical event occurred for the coronary event was 11.5 ± 1.9 years. Cox proportional hazards analysis showed that LTL was negatively associated with the incidences of coronary events and HF admission. Compared with the shortest quartile (0.38 per 100 PYRs), the incidence rate and adjusted risk of coronary events were significantly for the second (0.31 per 100 PYRs, HR: 0.93, 95% CI: 0.89–0.97, p < 0.001), third (0.26 per 100 PYRs, HR: 0.88, 95% CI: 0.84–0.92; p < 0.001), and longest quartiles (0.21 per 100 PYRs, HR: 0.83, 95% CI: 0.79–0.87; p < 0.001) (Fig. 3 and Supplementary Table 5).

**Figure 4:**
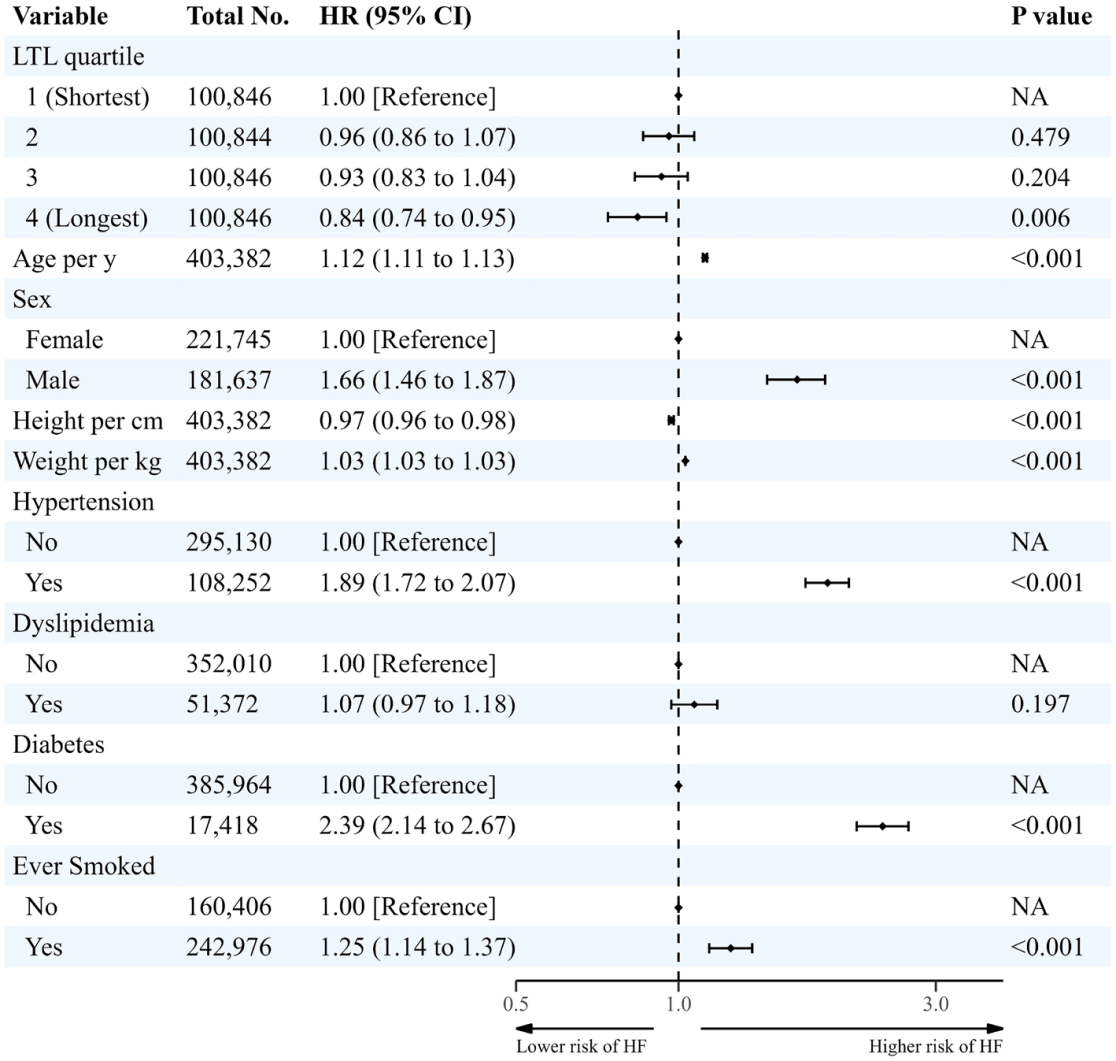
Longitudinal association between leukocyte telomere length and incidence of heart failure admission in white participants. *CI* confidence interval, *HF* heart failure, *HR* hazard ratio, *LTL* leukocyte telomere length, *NA* not applicable. Cox proportional hazards analysis was adjusted for age, sex, height, weight, hypertension, diabetes, dyslipidemia and ever smoked history.

The mean ± SD time to event when a medical event occurred for HF admission was 11.6 ± 1.6 years. The longest LTL quartile had a lower incidence of HF admission than shortest quartile (0.03 per 100 PYRs, HR: 0.84, 95% CI: 0.74–0.95; p = 0.006) (Fig. 4). Every 1 SD increase in LTL was associated with lower risks of coronary events (HR: 0.93, 95% CI: 0.91–0.95, p < 0.001) and HF admission (HR: 0.94, 95% CI: 0.90–0.98, p = 0.003) (Supplementary Table 5).

**Figure 5:**
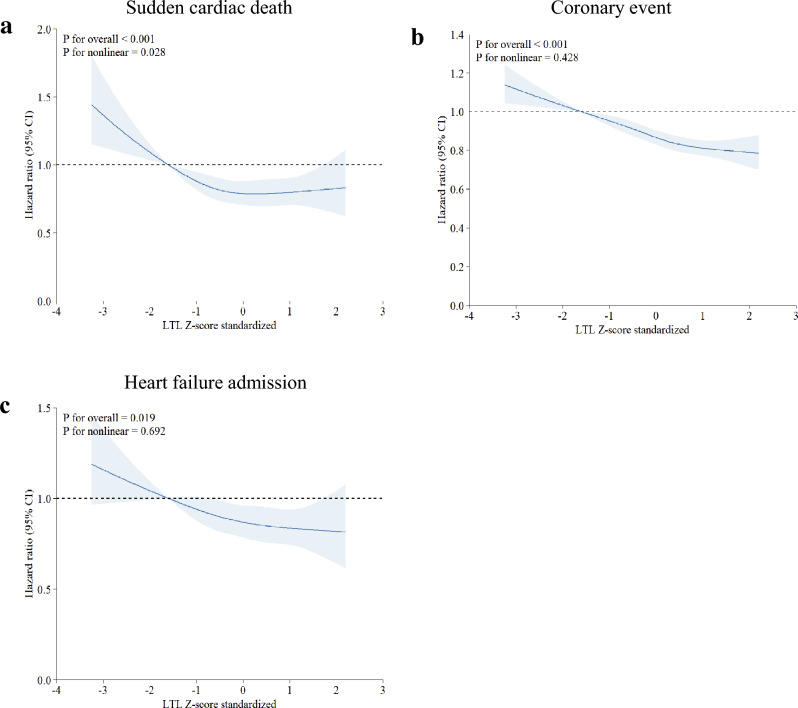
The dose–response associations of leukocyte telomere length with risk of incident sudden cardiac death (**a**), coronary event (**b**) and heart failure admission (**c**). *CI* confidence interval, *LTL* leukocyte telomere length. Restricted cubic spline models were fitted for Cox proportional hazard models, which were adjusted for age, sex, height, weight, hypertension, diabetes, dyslipidemia and ever smoked history.

Figure 5b shows the association between LTL and the risk of coronary events using the restricted cubic spline curve. The slope of the relationship was steep and linear (overall p-value < 0.001, nonlinear p-value = 0.428). HF admission risk and LTL had a similar association (overall p-value = 0.019, nonlinear p-value = 0.692) (Fig. 5c).

### Associations of LTL with CMR parameters and ECG parameters

After adjusting for clinical variables, LTL was independently positively associated with cardiac remodeling, namely LV mass index (β: 0.30, 95% CI: 0.21–0.38, p < 0.001), LV-end systolic (β: 0.28, 95% CI: 0.08–0.47; p = 0.005) and diastolic volumes (β: 0.57, 95% CI: 0.26–0.88, p < 0.001), mean LV myocardial wall thickness (β: 0.02, 95% CI: 0.01–0.03, p < 0.001), and LV stroke volume (β: 0.29, 95% CI: 0.09–0.49, p = 0.005) (Table 2).

**Table 2:** The associations of leukocyte telomere length with CMR parameters and ECG parameters in white participants by using linear regression models.

Intermediary cardiovascular phenotypes	β (95% CI)^a^	P value
CMR parameters		
LV related parameters		
LV-end diastolic volume, mL	0.57 (0.26 to 0.88)	< 0.001
LV-end systolic volume, mL	0.28 (0.08 to 0.47)	0.005
LV stroke volume, mL/beat	0.29 (0.09 to 0.49)	0.005
LVEF, %	− 0.03 (− 0.11 to 0.05)	0.431
LVMI, g/m^2^	0.30 (0.21 to 0.38)	< 0.001
Mean LV myocardial wall thickness, mm	0.02 (0.01 to 0.03)	< 0.001
LV strain		
LV circumferential global strain, %	0.02 (− 0.02 to 0.07)	0.263
LV radial global strain, %	0.07 (− 0.03 to 0.17)	0.182
LV longitudinal global strain, %	0.009 (− 0.03 to 0.05)	0.612
ECG parameters		
QRS duration, ms	0.18 (− 0.03 to 0.40)	0.090
QT interval, ms	0.28 (− 0.24 to 0.80)	0.296
QTc interval, ms	0.07 (− 0.33 to 0.47)	0.742
RR interval, ms	1.27 (− 1.43 to 3.98)	0.357
QRS-axis, degree	− 0.17 (− 0.73 to 0.38)	0.543
T-axis, degree	− 0.57 (− 1.07 to − 0.07)	0.027

*CI* confidence interval, *CMR* cardiovascular magnetic resonance, *ECG* electrocardiogram, *LV* left ventricle, *LVEF* left ventricular ejection fraction, *LVMI* left ventricular mass index.

^a^The analysis was adjusted for age, sex, height, weight, systolic blood pressure, diabetes, dyslipidemia, ever smoked history and total metabolic equivalent of task minutes per week.

After adjusting for clinical variables, there was no association between LTL and ECG parameters, namely QRS duration, QT interval, QTc interval, RR interval, and QRS-axis. However, it was associated with the degree of T-axis (β: − 0.57, 95% CI: − 1.07 to − 0.07, p = 0.027).

### Subgroup analysis

Subgroup analysis showed that the incidence ratios for the ≥ 65-years-old and < 65-years old groups were statistically significantly different (p for interaction 0.003) (Table 3). LTL was negatively associated with SCD risk regardless of gender or comorbidities.

**Table 3:** Subgroup analysis results for the association between leukocyte telomere length and sudden cardiac death.

Subgroup	HR (95% CI)^a^	P value	P value for interaction
Age			0.003
Age < 65 (N = 317,898)	0.98 (0.91 to 1.05)	0.516	
Age ≥ 65 (N = 85,484)	0.86 (0.78 to 0.94)	< 0.001	
Sex			0.760
Female (N = 221,745)	0.98 (0.89 to 1.09)	0.730	
Male (N = 181,637)	0.91 (0.85 to 0.97)	0.004	
Hypertension			0.434
No (N = 295,310)	0.91 (0.85 to 0.99)	0.019	
Yes (N = 108,252)	0.95 (0.87 to 1.03)	0.216	
Diabetes			0.313
No (N = 385,964)	0.92 (0.87 to 0.98)	0.007	
Yes (N = 17,418)	0.98 (0.83 to 1.15)	0.798	
Dyslipidemia			0.438
No (N = 352,010)	0.94 (0.89 to 1.00)	0.075	
Yes (N = 51,372)	0.88 (0.78 to 0.99)	0.028	
Atrial fibrillation			0.935
No (N = 399,313)	0.93 (0.88 to 0.98)	0.012	
Yes (N = 4069)	0.92 (0.69 to 1.22)	0.554	
CKD or ESRD			0.798
No (N = 400,379)	0.93 (0.88 to 0.98)	0.009	
Yes (N = 3003)	1.05 (0.72 to 1.54)	0.794	

*CI* confidence interval, *CKD* chronic kidney disease, *ESRD* end stage renal disease, *HR* hazard ratio.

^a^Multivariable Cox regression analysis was adjusted for age, sex, height, weight, hypertension, diabetes, dyslipidemia and ever smoked history. Hazard ratio indicates risk of sudden cardiac death per one Z-scores of the log_*e*_-transformed adjusted LTL T/S ratio.

## Discussion

This study’s results showed that LTL (1) was negatively associated with the incidences of SCD, coronary events and HF admission and this association was stronger in those 65 years old or older; (2) independently positively associated with cardiac remodeling, namely LV mass index, LV-end systolic and diastolic volumes, mean LV myocardial wall thickness, and LV stroke volume; and (3) negatively associated with T-axis degree.

### LTL association with SCD incidence

This cross-sectional analysis of white participants in the UK Biobank registry showed that LTL was negatively associated with the incidences of SCD, coronary events and HF admission. Participants were 40–69 years old and the two most common causes of SCD in this age group are coronary heart disease and cardiomyopathy, so we chose coronary events and HF admission as the secondary outcomes^23^. Our study showed similar results, which incident coronary event and HF admission were statistically significantly related to SCD. Interestingly, LTL was associated with SCD independent of incident coronary event and HF admission. Compared with the shortest quartile, adjusted SCD risk was significantly lower for the second and third quartiles. Telomere shortening might be related to atherosclerosis through various biological pathways^10^. Other SCD pathophysiology unrelated to atherosclerosis, such as arrhythmogenic disorder in structurally normal hearts, infiltrative heart diseases, and drug abuse, might have caused the longest quartile to have a higher adjusted SCD risk than the third quartile. LTL was negatively associated with SCD because it was negatively associated with incidences of coronary events and HF admission. However, nothing has been clearly revealed about independent associations of LTL with SCD which needs further research.

LTL and SCD were not statistically significantly associated among non-white participants (Supplementary Table 6). The small number of non-white participants may have limited our ability to detect statistically significant differences in the CI and HR for the association between LTL and SCD in non-white participants.

### The associations of LTL with heart size and cardiac function

We examined the association between LTL and intermediary cardiovascular phenotypes to determine why LTL was negatively associated with the incidences of SCD coronary events and HF admission. Intermediary cardiovascular phenotypes, such as coronary computed tomography angiography or echocardiography, were not included in the UK Biobank registry. Thus, we examined the association between LTL and other intermediary cardiovascular phenotypes, such as CMR and ECG parameters. LTL was positively associated with cardiac remodeling, including LV mass and LV stroke volume. LV loading is predominantly affected by LV volume, so LV has a similar pathophysiology as athletes’ heart^24,25^. Longer LTLs and higher stroke volumes increases positive ventricular loading, leading to higher LV mass and thickness. Similar findings about the association between LTL and LV mass were shown in previous studies^26,27^. This study’s CMR results showed that LTL was negatively associated with HF admission incidence in participants without a history of coronary heart disease or HF.

LV strain, myocardial deformation analysis, reflects ischemic damage and wall stress of myocardium. However, in this study, LTL was not associated with LV strain, namely LV circumferential global strain, LV radial global strain, and LV longitudinal global strain. In the UK Biobank registry, white participants first experienced coronary events onset 11.5 ± 1.9 years after their baseline visit. CMR was obtained 8.8 ± 1.7 years after their baseline visits, so no associations between LTL and LV strain parameters were acceptable, which requires further investigation.

Abnormal T-wave-axes indicate SCD^28^. LTL was negatively associated with SCD incidence, suggesting that LTL is negatively associated with T-wave-axes abnormality. LTL was negatively associated with T-wave-axis. However, considering Z-scores of the log_e_-transformed adjusted LTL T/S ratio ranges from − 3.2 to 2.2, the β coefficient of − 0.57 was too small to indicate that LTL is associated with T-wave-axis abnormality, so it may not explain SCD incidence. LTL was not associated with any ECG-related parameters, namely QRS duration, QT interval, QTc interval, RR interval, and QRS-axis.

### Limitations

This study’s first limitation is that it may have been subject to the healthy cohort bias because UK Biobank registry volunteers were more likely to be healthy and have fewer comorbidities than the general UK population. The second limitation is that CMR and ECG parameters were not obtained on the same date as LTL or when coronary events or HF admission occurred. The third limitation is that CMR and ECG were not done for every white participant. Even though we’ve adjusted age, sex, height, weight, systolic blood pressure, diabetes, dyslipidemia, ever smoked history and total metabolic equivalent of task minutes per week for obtaining associations of LTL with CMR and ECG parameters, participants who were involved in CMR and ECG work-up were more likely to be younger and male and less likely to have ever smoked history and cardiovascular or cerebrovascular comorbidities which can cause selection bias and limits generalizability (Supplementary Table 3 and Supplementary Table 4). The Fourth limitation is that there might be residual confounding variables. Due to observational nature, the current study cannot exclude residual confounding. The fifth limitation is the lack of multiple comparisons testing adjustment in order to adjust the p value related to associations of LTL with CMR and ECG parameters.

## Conclusion

This cross-sectional study showed that LTL was positively associated with heart size and better cardiac function in middle-aged participants which could explain the negative association between LTL and risk of SCD, coronary events, and HF admission in the UK Biobank participants. However, ECG results did not show these associations.

### Supplementary Information

Supplementary Tables.

## Data Availability

The data that support the findings of this study are available from UK Biobank but restrictions apply to the availability of these data, which were used under license for the current study, and so are not publicly available. Data are however available from the corresponding author upon reasonable request and with permission of UK Biobank.
