# Supplementary material for: The associations of leukocyte telomere length and intermediary cardiovascular phenotype with adverse cardiovascular outcomes in the white population
Source: Sci Rep. 2024 Jun 17;14:13975. doi: 10.1038/s41598-024-64997-3 (PMC11183248; doi:10.1038/s41598-024-64997-3)
Supplement: Supplementary file 1 — Supplementary Tables. [file 41598_2024_64997_MOESM1_ESM.docx]

**SUPPLEMENTARY MATERIAL**

*The Associations of Leukocyte Telomere Length and Intermediary Cardiovascular Phenotype with Adverse Cardiovascular Outcomes in the White Population*

Ho-Gi Chung, MD; Pil-Sung Yang, MD; Seunghoon Cho, MD; Eunsun Jang, MS; Daehoon Kim, MD; Hee Tae Yu, MD; Tae-Hoon Kim, MD; Jae-Sun Uhm, MD; Jung-Hoon Sung, MD; Hui-Nam Pak, MD; Moon-Hyoung Lee, MD; and Boyoung Joung, MD

| **Contents** | **Page** |
| --- | --- |
| **Supplementary Table 1.** STROBE Checklist for Cohort Study. | 2–5 |
| **Supplementary Table 2.** Definitions Used for Defining the Comorbidities. | 6–7 |
| **Supplementary Table 3.** Baseline Characteristics Between Non-CMR and CMR Group. | 8–9 |
| **Supplementary Table 4.** Baseline Characteristics Between Non-ECG and ECG Group. | 10–11 |
| **Supplementary Table 5.** The Association of Leukocyte Telomere Length with Risk of Sudden Cardiac Death, Coronary Event and Heart Failure Admission Using Cox Proportional Hazard Models. | 12 |
| **Supplementary Table 6.** Longitudinal Association Between Leukocyte Telomere Length and Incidence of Sudden Cardiac Death According to Race and Ethnicity. | 13 |

**Supplementary Table 1.** STROBE Checklist for Cohort Study.

|  | Item No. | Recommendation | Page  No. | Relevant text from manuscript |
| --- | --- | --- | --- | --- |
| **Title and abstract** | 1 | (*a*) Indicate the study’s design with a commonly used term in the title or the abstract | 1 | Abstract |
|  |  | (*b*) Provide in the abstract an informative and balanced summary of what was done and what was found | 1 | Abstract |
| Introduction | | | |  |
| Background/rationale | 2 | Explain the scientific background and rationale for the investigation being reported | 2 | Introduction: paragraph 1, 2 |
| Objectives | 3 | State specific objectives, including any prespecified hypotheses | 2 | Introduction: paragraph 3 |
| Methods | | | |  |
| Study design | 4 | Present key elements of study design early in the paper | 2, 3 | Methods: section 1 |
| Setting | 5 | Describe the setting, locations, and relevant dates, including periods of recruitment, exposure, follow-up, and data collection | 2, 3, 4 | Methods: section 1, 3 |
| Participants | 6 | (*a*) *Cohort study*—Give the eligibility criteria, and the sources and methods of selection of participants. Describe methods of follow-up | 2, 3, 4 | Methods: section 1, 3 |
|  |  | (*b*) *Cohort study*—For matched studies, give matching criteria and number of exposed and unexposed | Not applicable | Not applicable |
| Variables | 7 | Clearly define all outcomes, exposures, predictors, potential confounders, and effect modifiers. Give diagnostic criteria, if applicable | 3, 4, 5 | Methods: section 2, 3, 4, 5, 6 |
| Data sources/ measurement | 8 | For each variable of interest, give sources of data and details of methods of assessment (measurement). Describe comparability of assessment methods if there is more than one group | 3, 4, 5 | Methods: section 2, 3, 4, 5, 6 |
| Bias | 9 | Describe any efforts to address potential sources of bias | 3 | Methods: section 1 |
| Study size | 10 | Explain how the study size was arrived at | 3 | Methods: section 1 |

| Quantitative variables | 11 | Explain how quantitative variables were handled in the analyses. If applicable, describe which groupings were chosen and why | 3, 4, 5 | Methods: section 2, 4, 5 |
| --- | --- | --- | --- | --- |
| Statistical methods | 12 | (*a*) Describe all statistical methods, including those used to control for confounding | 5, 6 | Methods: section 6 |
|  |  | (*b*) Describe any methods used to examine subgroups and interactions | 5 | Methods: section 6 |
|  |  | (*c*) Explain how missing data were addressed | 6 | Methods: section 6 |
|  |  | (*d*) *Cohort study*—If applicable, explain how loss to follow-up was addressed | 3 | Methods: section 1 |
|  |  | (*e*) Describe any sensitivity analyses | 5 | Methods: section 6 |
| Results | | | | |
| Participants | 13* | (a) Report numbers of individuals at each stage of study—eg numbers potentially eligible, examined for eligibility, confirmed eligible, included in the study, completing follow-up, and analysed | 3 | Results: section 1 |
|  |  | (b) Give reasons for non-participation at each stage | 3 | Methods: section 1 |
|  |  | (c) Consider use of a flow diagram | 3 | Figure 1 |
| Descriptive data | 14* | (a) Give characteristics of study participants (eg demographic, clinical, social) and information on exposures and potential confounders | 6 | Results: section 1, Table 1 |
|  |  | (b) Indicate number of participants with missing data for each variable of interest | 6 | Methods: section 6 |
|  |  | (c) *Cohort study*—Summarise follow-up time (eg, average and total amount) | 6, 7, 8 | Results: section 2, 3 |
| Outcome data | 15* | *Cohort study*—Report numbers of outcome events or summary measures over time | 6, 7, 8, 9 | Results: section 2, 3, 4, 5 |
| Main results | 16 | (*a*) Give unadjusted estimates and, if applicable, confounder-adjusted estimates and their precision (eg, 95% confidence interval). Make clear which confounders were adjusted for and why they were included | 6, 7, 8, 9 | Results: section 2, 3, 4, 5, Figure 2, 3, 4, 5 |
|  |  | (*b*) Report category boundaries when continuous variables were categorized | 6, 7, 8, 9 | Results: section 2, 3, 4, 5 |

Continued on next page

| Other analyses | 17 | Report other analyses done—eg analyses of subgroups and interactions, and sensitivity analyses | 7, 9 | Results: section 2, Table 2 |
| --- | --- | --- | --- | --- |
| Discussion | | | | |
| Key results | 18 | Summarise key results with reference to study objectives | 9 | Discussion: paragraph 1 |
| Limitations | 19 | Discuss limitations of the study, taking into account sources of potential bias or imprecision. Discuss both direction and magnitude of any potential bias | 11 | Limitations |
| Interpretation | 20 | Give a cautious overall interpretation of results considering objectives, limitations, multiplicity of analyses, results from similar studies, and other relevant evidence | 9, 10, 11 | Discussion |
| Generalisability | 21 | Discuss the generalisability (external validity) of the study results | 11 | Limitations |
| Other information | |  | | |
| Funding | 22 | Give the source of funding and the role of the funders for the present study and, if applicable, for the original study on which the present article is based | 16 | Funding section |

**Supplementary Table 2.** Definitions Used for Defining the Comorbidities.

| **Comorbidities** | **UK Biobank** | |
| --- | --- | --- |
|  | **Definitions** | **Used codes or conditions** |
| Atrial fibrillation | Defined from U.K. Biobank self-report or diagnosis^a^ | Self-reported non-cancer illness code: 1471, 1483  ICD-10: I48 |
| Heart failure | Defined from U.K. Biobank self-report or diagnosis^a^ | Self-reported non-cancer illness code: 1076  ICD-10: I11.0, I50, I97.1 |
| Ischemic stroke | Defined from U.K. Biobank self-report or diagnosis^a^ | Self-reported non-cancer illness code: 1583  ICD-10: I63, I64 |
| TIA | Defined from U.K. Biobank self-report or diagnosis^a^ | Self-reported non-cancer illness code: 1082  ICD-10: G45 |
| Diabetes mellitus | Defined from U.K. Biobank self-report or diagnosis^a^ | Self-reported non-cancer illness code: 1220, 1222, 1223, 1521  ICD-10: E10, E11, E12, E13, E14 |
| Hypertension | Defined from U.K. Biobank self-report or diagnosis^a^ | Self-reported non-cancer illness code: 1065, 1072  ICD-10: I10, I11, I12, I13, I15 |
| Previous myocardial infarction (MI) | Defined from U.K. Biobank self-report or diagnosis^a^ | Self-reported non-cancer illness code: 1075  ICD-10: I21, I22, I25.2 |
| Peripheral arterial disease | Defined from U.K. Biobank self-report or diagnosis^a^ | Self-reported non-cancer illness code: 1067, 1087  ICD-10: I70, I71 |
| Dyslipidemia | Defined from U.K. Biobank self-report or diagnosis^a^ | Self-reported non-cancer illness code: 1473  ICD-10: E78 |
| COPD | Defined from U.K. Biobank self-report or diagnosis^a^ | Self-reported non-cancer illness code: 1112, 1113, 1472  ICD-10: J42, J43(except J43.0), J44 |
| Chronic renal failure | Defined from eGFR (if laboratory value was not available, self-report or diagnosis code was used) | eGFR <60 mL/min per 1.73 m^2^  Self-reported non-cancer illness code:1192, 1194  ICD-10: N18, N19 |
| ESRD | Defined from U.K. Biobank self-report or procedure codes related to renal replacement therapy (hemodialysis, peritoneal dialysis, or kidney transplant) | Self-reported non-cancer illness code: 1193, 1195, 1580, 1581, 1582  Procedure codes: L74, M01, M02.3, M08.4, M17, X40, X41, X42 |

^a^To ensure accuracy, comorbidities were established based on more than one hospital-inpatient or two outpatients (=primary care in United Kingdom) records of ICD-10 codes in the database.

**Supplementary Table 3.** Baseline Characteristics Between Non-CMR and CMR Group.

| Cohort characteristics | Full cohort | Non-CMR  (N=370,339) | CMR  (N=33,043) | P value |
| --- | --- | --- | --- | --- |
| Age at telomere visit, y | 57.1 ± 8.0 | 57.3 ± 8.1 | 55.5 ± 7.4 | <0.001 |
| LTL quartile, No. (%) |  |  |  | <0.001 |
| 1^st^ | 100,846 (25.0) | 93,345 (25.2) | 7,501 (22.7) |  |
| 2^nd^ | 100,844 (25.0) | 92,620 (25.0) | 8,224 (24.9) |  |
| 3^rd^ | 100,846 (25.0) | 92,443 (25.0) | 8,403 (25.4) |  |
| 4^th^ | 100,846 (25.0) | 91,931 (24.8) | 8,915 (27.0) |  |
| Sex, No. (%) |  |  |  | <0.001 |
| Female | 221,745 (55.0) | 204,423 (55.2) | 17,322 (52.4) |  |
| Male | 181,637 (45.0) | 165,916 (44.8) | 15,721 (47.6) |  |
| Height, cm | 168.7 ± 9.3 | 168.6 ± 9.3 | 169.8 ± 9.2 | <0.001 |
| Weight, kg | 77.9 ± 15.8 | 78.0 ± 15.9 | 76.7 ± 14.7 | <0.001 |
| Systolic BP, mmHg | 138.2 ± 18.7 | 138.5 ± 18.8 | 135.4 ± 17.8 | <0.001 |
| Diastolic BP, mmHg | 82.4 ± 10.1 | 82.4 ± 10.2 | 81.5 ± 9.9 | <0.001 |
| Heart rate, beats/min | 69.7 ± 11.6 | 69.8 ± 11.7 | 68.0 ± 10.9 | <0.001 |
| Hypertension, % | 26.8 | 27.5 | 19.3 | <0.001 |
| Diabetes, % | 4.3 | 4.5 | 2.3 | <0.001 |
| Dyslipidemia, % | 12.7 | 13.0 | 9.3 | <0.001 |
| Ever smoked, % | 60.2 | 60.7 | 57.3 | <0.001 |
| Physical activity (total MET min per wk) | 2678.8 ± 2722.2 | 2699.4 ± 2748.2 | 2461.9 ± 2422.9 | <0.001 |
| WBC, count/μL | 6900 ± 2000 | 6900 ± 2000 | 6600 ± 1800 | <0.001 |
| Ischemic stroke or TIA, % | 0.7 | 0.8 | 0.4 | <0.001 |
| PAOD, % | 0.2 | 0.2 | 0.1 | <0.001 |
| COPD, % | 0.2 | 0.2 | 0.1 | <0.001 |
| CKD or ESRD, % | 0.7 | 0.8 | 0.4 | <0.001 |
| AF, % | 1.0 | 1.0 | 0.6 | <0.001 |
| VT history, % | 0.2 | 0.3 | 0.1 | <0.001 |
| BP medication, % | 18.9 | 19.4 | 12.6 | <0.001 |
| Statin, % | 13.4 | 13.7 | 9.2 | <0.001 |
| Antiplatelet, % | 11.9 | 12.1 | 9.5 | <0.001 |

Values are presented as mean **±** standard deviation or number (%).

Abbreviations: AF, atrial fibrillation; BP, blood pressure; CKD, chronic kidney disease; CMR, cardiovascular magnetic resonance imaging; COPD, chronic obstructive pulmonary disease; ESRD, end stage renal disease; LTL, leukocyte telomere length; LV, left ventricle; LVEF, left ventricle ejection fraction; LVMI, left ventricle mass index; MET, metabolic equivalent of task; NA, not applicable; PAOD, peripheral artery occlusive disease; SD, standard deviation, TIA, transient ischemic attack; VT, ventricular tachycardia; WBC, white blood cell.

**Supplementary Table 4.** Baseline Characteristics Between Non-ECG and ECG Group.

| Cohort characteristics | Full cohort | Non-ECG  (N=383,828) | ECG  (N=19,554) | P value |
| --- | --- | --- | --- | --- |
| Age at telomere visit, y | 57.1 ± 8.0 | 57.2 ± 8.0 | 55.5 ± 7.5 | <0.001 |
| LTL quartile, No. (%) |  |  |  | <0.001 |
| 1^st^ | 100,846 (25.0) | 96,266 (25.1) | 4,580 (23.4) |  |
| 2^nd^ | 100,844 (25.0) | 95,959 (25.0) | 4,885 (25.0) |  |
| 3^rd^ | 100,846 (25.0) | 95,937 (25.0) | 4,909 (25.1) |  |
| 4^th^ | 100,846 (25.0) | 95,666 (24.9) | 5,180 (26.5) |  |
| Sex, No. (%) |  |  |  | <0.001 |
| Female | 221,745 (55.0) | 211,564 (55.1) | 10,181 (52.1) |  |
| Male | 181,637 (45.0) | 172,264 (44.9) | 9,373 (47.9) |  |
| Height, cm | 168.7 ± 9.3 | 168.6 ± 9.3 | 169.7 ± 9.2 | <0.001 |
| Weight, kg | 77.9 ± 15.8 | 78.0 ± 15.8 | 77.0 ± 14.9 | <0.001 |
| Systolic BP, mmHg | 138.2 ± 18.7 | 138.4 ± 18.8 | 135.6 ± 17.7 | <0.001 |
| Diastolic BP, mmHg | 82.4 ± 10.1 | 82.4 ± 10.2 | 81.6 ± 9.9 | <0.001 |
| Heart rate, beats/min | 69.7 ± 11.6 | 69.8 ± 11.6 | 68.0 ± 10.9 | <0.001 |
| Hypertension, % | 26.8 | 27.2 | 19.9 | <0.001 |
| Diabetes, % | 4.3 | 4.4 | 2.4 | <0.001 |
| Dyslipidemia, % | 12.7 | 12.9 | 9.7 | <0.001 |
| Ever smoked, % | 60.2 | 60.6 | 57.1 | <0.001 |
| Physical activity (total MET min per wk) | 2678.8 ± 2722.2 | 2688.5 ± 2734.3 | 2498.7 ± 2482.9 | <0.001 |
| WBC, count/μL | 6900 ± 2000 | 6900 ± 2000 | 6600 ± 1900 | <0.001 |
| Ischemic stroke or TIA, % | 0.7 | 0.8 | 0.4 | <0.001 |
| PAOD, % | 0.2 | 0.2 | 0.1 | <0.001 |
| COPD, % | 0.2 | 0.2 | 0.1 | <0.001 |
| CKD or ESRD, % | 0.7 | 0.8 | 0.5 | <0.001 |
| AF, % | 1.0 | 1.0 | 0.5 | <0.001 |
| VT history, % | 0.2 | 0.2 | 0.1 | 0.001 |
| BP medication, % | 18.9 | 19.2 | 13.1 | <0.001 |
| Statin, % | 13.4 | 13.5 | 9.6 | <0.001 |
| Antiplatelet, % | 11.9 | 12.0 | 9.5 | <0.001 |

Values are presented as mean **±** standard deviation or number (%).

Abbreviations: AF, atrial fibrillation; BP, blood pressure; CKD, chronic kidney disease; COPD, chronic obstructive pulmonary disease; ECG, electrocardiogram; ESRD, end stage renal disease; LTL, leukocyte telomere length; LV, left ventricle; LVEF, left ventricle ejection fraction; LVMI, left ventricle mass index; MET, metabolic equivalent of task; NA, not applicable; PAOD, peripheral artery occlusive disease; SD, standard deviation, TIA, transient ischemic attack; VT, ventricular tachycardia; WBC, white blood cell.

**Supplementary Table 5.** The Association of Leukocyte Telomere Length with Risk of Sudden Cardiac Death, Coronary Event and Heart Failure Admission Using Cox Proportional Hazard Models.

| Primary and secondary outcomes | Event, n | Event per 100 PYRs | HR (95% CI) ^a^ | P value |
| --- | --- | --- | --- | --- |
| Sudden cardiac death |  |  |  |  |
| LTL quartile † |  |  |  |  |
| 1 (Shortest) | 629 | 0.05 | 1.00 [Reference] | NA |
| 2 | 457 | 0.04 | 0.82 (0.73 to 0.93) | 0.002 |
| 3 | 409 | 0.03 | 0.81 (0.72 to 0.92) | 0.001 |
| 4 | 394 | 0.03 | 0.90 (0.79 to 1.02) | 0.097 |
| Per 1-SD increase | 1,889 | 0.04 | 0.92 (0.88 to 0.97) | <0.001 |
| Coronary event |  |  |  |  |
| LTL quartile † |  |  |  |  |
| 1 (Shortest) | 4,398 | 0.38 | 1.00 [Reference] | NA |
| 2 | 3,570 | 0.31 | 0.93 (0.89 to 0.97) | <0.001 |
| 3 | 3,029 | 0.26 | 0.88 (0.84 to 0.92) | <0.001 |
| 4 | 2,473 | 0.21 | 0.83 (0.79 to 0.87) | <0.001 |
| Per 1-SD increase | 13,470 | 0.29 | 0.93 (0.91 to 0.95) | <0.001 |
| Heart failure admission |  |  |  |  |
| LTL quartile † |  |  |  |  |
| 1 (Shortest) | 764 | 0.07 | 1.00 [Reference] | NA |
| 2 | 624 | 0.05 | 0.96 (0.86 to 1.07) | 0.479 |
| 3 | 530 | 0.04 | 0.93 (0.83 to 1.04) | 0.204 |
| 4 | 398 | 0.03 | 0.84 (0.74 to 0.95) | 0.006 |
| Per 1-SD increase | 2,316 | 0.05 | 0.94 (0.90 to 0.98) | 0.003 |

Abbreviations: CI, confidence interval; HR, hazard ratio; LTL, leukocyte telomere length; NA, not applicable; PYRs, person-years; SD, standard deviation.

^a^ Multivariable Cox regression analysis was adjusted for age, sex, height, weight, hypertension, diabetes, dyslipidemia and ever smoked history.

† LTL was categorized according to the quartile of T/S ratio.

**Supplementary Table 6.** Longitudinal Association Between Leukocyte Telomere Length and Incidence of Sudden Cardiac Death According to Race and Ethnicity.

| Sudden cardiac death | HR (95% CI) ^a^ | P value |
| --- | --- | --- |
| Race and ethnicity |  |  |
| Asian or Asian British (N=7,875) | 1.01 (0.72 to 1.41) | 0.972 |
| Black or Black British (N=2,762) | 1.01 (0.55 to 1.84) | 0.983 |
| Chinese (N=1,308) | 1.08 (0.24 to 4.84) | 0.917 |
| White (N=403,382) | 0.92 (0.87 to 0.97) | <0.001 |
| Mixed race (N=2,493) | 1.40 (0.70 to 2.81) | 0.340 |
| Other ethnic group (N=3,744) | 0.68 (0.36 to 1.28) | 0.229 |

Abbreviations: CI, confidence interval; HR, hazard ratio.

^a^ Multivariable Cox regression analysis was adjusted for age, sex, height, weight, hypertension, diabetes, dyslipidemia and ever smoked history. Hazard ratio indicates risk of sudden cardiac death per one Z-scores of the log*_e_*-transformed adjusted LTL T/S ratio.
